# Drug Users in Amsterdam: Are They Still at Risk for HIV?

**DOI:** 10.1371/journal.pone.0059125

**Published:** 2013-03-18

**Authors:** Nienke van der Knaap, Bart P. X. Grady, Maarten F. Schim van der Loeff, Titia Heijman, Arjen Speksnijder, Ronald Geskus, Maria Prins

**Affiliations:** 1 Cluster of Infectious Diseases, Public Health Service, Amsterdam, The Netherlands; 2 University of Amsterdam (UvA), Amsterdam, The Netherlands; 3 Center of Infectious diseases and Immunology Amsterdam (CINIMA), Department of Internal Medicine, Academic Medical Center (AMC), Amsterdam, The Netherlands; 4 Laboratory of Public Health, Public Health Service, Amsterdam, The Netherlands; 5 Department of Internal Medicine, Division of Infectious Diseases, Tropical Medicine and AIDS, Academic Medical Center, Amsterdam, The Netherlands; 6 Department of Clinical Epidemiology, Biostatistics and Bioinformatics, Academic Medical Center (AMC), Amsterdam, The Netherlands; California Pacific Medicial Center Research Institute, United States of America

## Abstract

**Background and Aims:**

To examine whether drug users (DU) in the Amsterdam Cohort Study (ACS) are still at risk for HIV, we studied trends in HIV incidence and injecting and sexual risk behaviour from 1986 to 2011.

**Methods:**

The ACS is an open, prospective cohort study on HIV. Calendar time trends in HIV incidence were modelled using Poisson regression. Trends in risk behaviour were modelled via generalized estimating equations. In 2010, a screening for STI (chlamydia, gonorrhoea and syphilis) was performed. Determinants of unprotected sex were studied using logistic regression analysis.

**Results:**

The HIV incidence among 1298 participants of the ACS with a total follow-up of 12,921 person-years (PY) declined from 6.0/100 PY (95% confidence interval [CI] 3.2–11.1) in 1986 to less than 1/100 PY from 1997 onwards. Both injection and sexual risk behaviour declined significantly over time. Out of 197 participants screened for STI in 2010–2011, median age 49 years (IQR 43–59), only 5 (2.5%) were diagnosed with an STI. In multivariable analysis, having a steady partner (aOR 4.1, 95% CI 1.6–10.5) was associated with unprotected sex. HIV-infected participants were less likely to report unprotected sex (aOR 0.07, 95% CI 0.02–0.37).

**Conclusions:**

HIV incidence and injection risk behaviour declined from 1986 onwards. STI prevalence is low; unprotected sex is associated with steady partners and is less common among HIV-infected participants. These findings indicate a low transmission risk of HIV and STI, which suggests that DU do not play a significant role in the current spread of HIV in Amsterdam.

## Introduction

Drug users (DU) are at risk for HIV through both injection risk behaviour and sexual risk behaviour, with injection risk behaviour being the predominant mode of HIV transmission [1]. Injecting drug users account for 22% of all newly diagnosed HIV infections in Europe [2]. However, sexual risk behaviour seems to play an increasing role in the acquisition of HIV among DU [3]–[7].

In addition to the separate patterns of transmission, there is a degree of overlap between (injecting) drug use and sexual risk behaviour. Use of stimulants (e.g., cocaine) is associated with an increased risk of unprotected sex [1], [8], [9]. Another overlap was seen in DU who worked as commercial sex workers (CSW) [10], [11]. DU who are involved in sex work show higher rates of unprotected sex and sexually transmitted infections (STI) [3], [12].

It has been suggested that comprehensive harm-reduction programmes contributed to the stabilisation or even decline of HIV incidence among DU in the Netherlands and other countries in Western Europe and North America [13]–[15]. However, harm-reduction programmes are usually directed at reducing injection risk behaviour and less at reducing sexual risk behaviour [16].

Between 1986 and 2005, we observed a decline in injecting risk behaviour among DU in Amsterdam. However, sexual risk behaviour remained substantial in this study and was considered to be the main risk factor for HIV acquisition [7]. In order to examine whether DU are still at risk for HIV, we updated analyses for trends in HIV incidence, and both injection and sexual risk behaviour in the Amsterdam Cohort Study (ACS) among DU from 1986 until 2011. In addition, to assess whether there is a need for interventions to reduce sexual risk behaviour, we tested for STIs and examined determinants for unprotected sex among those study participants who had a study visit in 2010 to 2011.

## Materials and Methods

### Ethics Statement

The medical ethics committee of the Academic Medical Center (MEC AMC, 09/40) approved the current observational study. Enrolment is voluntary, anonymous and written informed consent was obtained from each participant at intake visit. Participants agreed that questionnaires and blood samples or other specimens will be used for research purposes. Participants can withdraw from the study at anytime. All potential participants who declined or did not participate, were not disadvantaged in any other way by not participating in this study.

### Study Population

In December 1985, the ACS among DU (www.amsterdamcohortstudies.org) was initiated [17]. Recruitment is still ongoing and in recent years has been directed in particular to young DU. Over time, an estimated 15% of the Amsterdam injecting DU population participated in the ACS [18]. Participants return for follow-up visits every 4 (until 2003) to 6 months. At each visit, trained research nurses interview the participants regarding sociodemographic information, (injecting) drug use, sexual risk behaviour and STIs, using a standardized questionnaire. Questions at study entry refer to the preceding 6 months, questions during follow-up refer to the period between the current and the previous visit. In addition, at every visit blood is collected for storage and to test for HIV antibodies.

We offered STI screening to all participants of the ACS who had a cohort visit between November 2010 and June 2011. Participants who consented to STI screening are further referred to as ‘recent visitors’. These recent visitors were tested for *Chlamydia trachomatis* (CT), *Neisseria gonorrhoeae* (NG) and *Trepanoma pallidum* (syphilis). Self-collected urine samples (males) and self-collected vaginal swabs (females) were used to test for CT and NG. Serum was tested for syphilis. Participants at high risk for STI were not tested at the cohort study clinic but were directly referred to the outpatient STI clinic of the Public Health Service of Amsterdam in the same building [19]. High risk for STI was defined as: receiving money for sex in the previous 6 months and not being tested for STI during the last 6 months; symptoms suggestive of an STI; participants who were notified about STI exposure by their sexual partner(s); and (for male participants only) having had sex with men in the previous 6 months.

### Laboratory Methods

At each visit, serum was tested for HIV antibodies (Ag/Ab Combo test, Axsym; Abbott Laboratories and bioMérieux, France). Reactive samples were confirmed by immunoblot (Line Immuno Assay, Inno-Lia HIV I/II Score; Innogenetics NV, Gent, Belgium). Before 2004, reactive samples were confirmed by Western blot.

For the recent visitors, self-collected urine (male) or vaginal swab (female) samples were tested for CT and NG using Nucleic Acid Amplification Test (NAAT) (Gen-probe Aptima Combo 2 Assay, San Diego, CA, USA). Serum was tested for syphilis (*Treponema pallidum* particle agglutination assay; Serodia-TPPA; Fujirebio Europe BV). To confirm and classify syphilis, reactive samples were further tested by the Venereal Disease Research Laboratory (VDRL) test (Wellcome, Dartford, UK), RPR-nosticon II (rapid plasma reagin; Biomérieux, Marcy l’Etoile, France) and the FTA-absorption test (Trepo-Spot IF; Biomérieux, Marcy l’Etoile, France). Positive test results were classified into ‘infectious syphilis’ (TPPA ≥1∶80 and VDRL ≥1∶8) and ‘previously treated syphilis’ (TPPA ≥1∶80 and VDRL >1∶1).

Laboratory procedures for high-risk participants have been described elsewhere [20]. In brief, urine samples and vaginal swabs were tested for CT and NG. Serum was tested for syphilis. Direct microscopy on gram stains and wet mounts was performed for NG, non-specific urethritis (NSU) in male participants and NG and *Trichomonas vaginalis* (TV) in female participants.

### STI Treatment

Participants of the STI screening received negative test results by letter. In the event of positive test results, participants were seen at the study site by the physician of the ACS and subsequently received treatment under supervision of dermatologists of our STI clinic.

### Statistical Analysis

To investigate trends in HIV incidence among all HIV-negative DU at ACS entry, the observed HIV incidence rate per calendar year was calculated using person-time techniques. The date of HIV seroconversion was estimated as the midpoint between the last HIV-seronegative and the first HIV-seropositive ACS visit. Trends in HIV incidence rates over calendar time among all DU and injecting DU were modelled separately.

Among HIV-negative participants, trends in self-reported injecting and sexual risk behaviour, use of needle exchange and STI were modelled using logistic regression. Adjusting for multiple visits per individual was done by using generalised estimating equations assuming an exchangeable working covariance matrix. Information regarding unprotected sex was available from 1991 onwards. We defined unprotected sex as not or not always having used a condom while practising vaginal or anal sex. Trends and HIV incidence rates over calendar time were allowed to vary smoothly using natural cubic splines [21].

To examine determinants of unprotected sex among the recent visitors, logistic regression was used. Participants who did not report vaginal or anal sex in the preceding 6 months were excluded from this analysis. Potential determinants included variables of sociodemographics, drug use and sexual behaviour. Unprotected sex was reported separately for each partner type from 2009 onwards. We distinguished steady partner, casual partner, client and CSW. To account for participants who reported multiple partner types, generalised estimating equations (GEE) were used [22]. A multivariable model was built including variables with a univariable p-value <0.20 after which backward stepwise selection was used with a p-value of 0.05. A p-value <0.05 was considered statistically significant.

Statistical analysis was performed by use of SPSS software (version 19.0; SPSS Inc.) and the R statistical computing environment (version 2.14.0; http://www.R-project.org/).

## Results

### Demographics

From December 1985 until December 2011, 1658 DU had been enrolled in the ACS, of whom 1298 DU had at least 2 visits with a total follow-up of 12,921 person years. The median age at entry was 30 years (IQR 26–35), 62% were male and 71% had Dutch nationality, see table 1. Of all the DU, 1158 (69%) participants reported at baseline that they had ever injected drugs and 59 participants started injecting drugs during follow-up. The median follow-up time was 9.2 years (IQR 3.7–14.8). By 31 December 2011, 464 DU had died. The yearly return rate, defined as participants who visited the ACS during a given calendar year and returned for a follow-up visit the next year, was 94% (IQR 92–96) and was stable over time.

**Table 1:** Characteristics of the recent visitors of the Amsterdam Cohort Studies who participated in an STI screening (N = 197) between 2010–2011 and all visitors (N = 1658) at baseline between December 1985 and 2011.

	Recent visitors	All visitors
	N = 197	N = 1658
Gender		
Male	141 (72%)	1035 (62%)
Nationality		
Dutch	166 (84%)	1183 (71%)
Non-Dutch	31 (16%)	475 (29%)
Age, median (IQR)	49 (43–54)	30 (26–35)
HIV		
Positive at study entry	15 (8%)	322 (19%)
Housing situation		
Supervised housing^a^	86 (44%)	359 (28%)
Having a steady partner		
Yes	69 (35%)	755 (46%)
Partner type b		
None	90 (46%)	*
Steady	62 (32%)	*
Casual	42 (21%)	*
Client	9 (5%)	*
CSW	14 (7%)	*
Sexual risk behaviour#		
No	125 (64%)	511 (56%)
Yes, unprotected^c^	72 (36%)	294 (44%)
Ever injected drugs		
Yes	124 (63%)	1158 (69%)
Injected drugs in past6 months		
Yes	23 (12%)	868 (52%)
Drug use in past 6 months^d^		
Only cocaine	142 (72%)	559 (34%)
Only heroin	99 (50%)	702 (42%)
Cocktail^e^	7 (4%)	383 (23%)
Cannabis	115 (58%)	*
Other^f^	92 (47%)	295 (18%)
Alcohol (any daily)		
Yes	116 (59%)	333 (20%)
Methadone prescription		
Yes	123 (62%)	636 (38%)

CSW, commercial sex worker; IQR, interquartile range; STI, sexually transmitted infection.

a Supervised: living in a hotel/pension, institutional care, lodging.

b Partner type: per individual more than 1 partner type possible.

c Unprotected sex: Inconsistent condom use.

d Includes injecting and noninjecting drug use; per individual more than 1 type of drug use possible.

e Heroin and cocaine together.

f Includes benzodiazepines, amphetamines and barbiturates.

# Questions on sexual risk behaviour were available from 1991 onwards.

* Questions on partner types and cannabis use were only available from 2009 onwards.

### HIV Incidence

**Figure 1:**
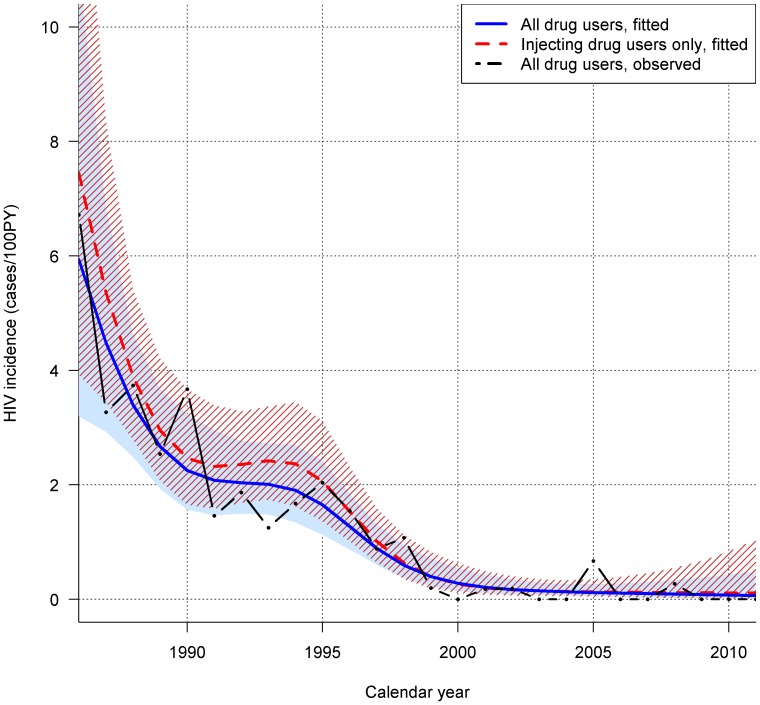
Observed and fitted HIV incidence in the Amsterdam Cohort Studies among drug users (DU), for all DU and for injecting DU only, 1986–2011. The shaded and striped areas represent the 95% confidence intervals for, respectively, all DU (fitted) and injecting DU only (fitted).

Out of 1298 DU with at least two visits, 261 (20%) participants were HIV-infected at study entry and 97 participants seroconverted for HIV during follow-up. Median age at HIV seroconversion was 33 years (IQR 28–38), 59% were male and 80% had Dutch nationality. The number of HIV-negative DU in follow-up increased from 133 DU in 1986 to 598 in 2001, and then declined to 284 in 2011. Figure 1 shows the observed HIV incidence for all DU and the fitted HIV incidence for all DU and injecting DU only. The fitted HIV incidence rate among all DU of the ACS was initially high: 5.96/100 PY (95% confidence interval (CI) 3.21–11.05) in 1986, but decreased significantly over time (p<0.001) and the fitted HIV incidence remained less than 1/100 PY from 1997 onwards. For injectors only, the fitted HIV incidence was slightly higher at 7.47/100 PY (95% CI 3.94–14.16) in 1986, decreasing over time (p<0.001) to less than 1/100 PY from 1997 onwards.

### Injecting Drug Use and Sexual Risk Behaviour

**Figure 2:**
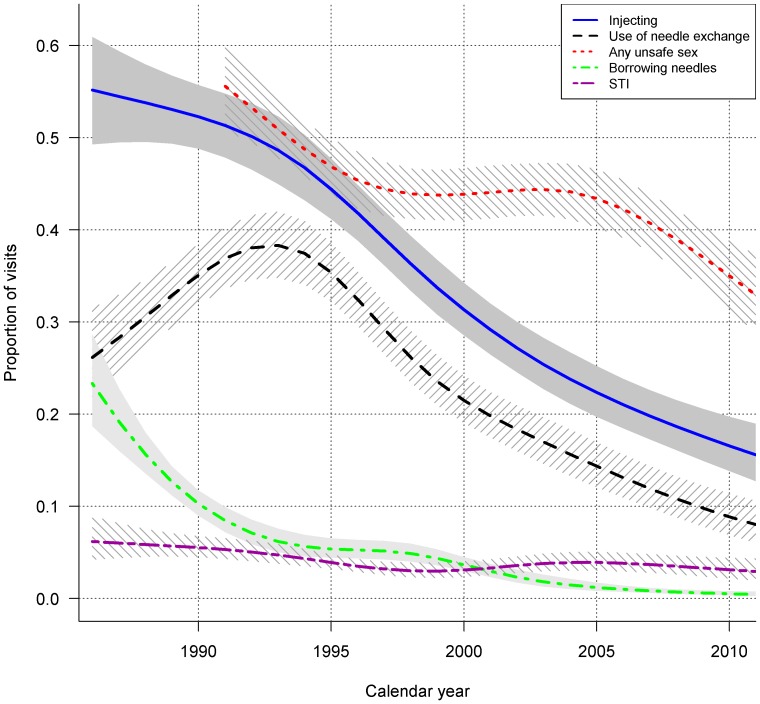
Fitted trends in self-reported injecting and sexual risk behaviour of proportion of visits per calendar year among DU of the Amsterdam Cohort Studies, 1986–2011. The shaded and striped and grey areas represent the 95% confidence intervals. STI, sexual transmitted infection.

Estimated trends in injecting drug use and sexual risk behaviour from 1986 till 2010 are shown in figure 2. Although there was some variation over time, injecting risk behaviour and use of needle exchange showed declining trends. The prevalence of injecting drug use decreased drastically over time, as did the prevalence of borrowing needles (both test for trend p<0.001). The use of needle exchange increased up to 38.2% (95% CI 34.8–41.6) in 1993 and decreased over time to 8.5% (95% CI 6.5–11.1) in 2010 (test for trend p<0.001). Of interest, this decrease occurred at a comparable rate as the prevalence of reported injecting. The prevalence of any unsafe sex was 55.6% (95% CI 50.8–60.2) in 1991 and decreased to 45.4% (95% CI 42.3–48.5) in 1996. Between 1997 and 2005 the trend appeared relatively stable over time, respectively 44.4% (41.5–47.3) and 43.4% (95% CI 40.3–46.6). After 2005, the prevalence of any unsafe sex declined to 35.0% (95% CI 31.4–38.8) in 2010, test for trend p<0.001. The prevalence of self-reported STIs was 6.3% (95% CI 4.3–9.2) in 1986 and decreased to 3.1% (95% CI 2.6–4.6) in 2010, test for trend p = 0.011.

A decline in risk behaviour during 25 years of observation could be caused by drop out or death of participants with high-risk behaviour. In a sensitivity analysis, we selected participants with a study visit in 2010. The trends of observed proportions of visits with self-reported STIs, any unprotected sex, injecting and borrowing of needles in the group with a 2010 visit were comparable to those in the total group. On average, injecting drug use was lower in the group with a visit in 2010 when compared to the total group, although this difference became less apparent since 1997. Use of needle exchange also started lower in the group with a 2010 visit, but followed the same decline over time (data not shown).

To rule out a cohort effect, we censored follow-up 3 years after study entry. Trends over time were comparable, although we observed no decline in any unsafe sex after 2005.

### STI and Unprotected Sex Among the Recent Visitors

Between November 2010 and June 2011, 272 individuals had a cohort visit, of whom 197 (72.4%) underwent an STI screening. Of the visitors who were not willing to participate in this STI screening, 64.0% indicated that they had no personal interest, 17.3% were recently tested, and 9.3% did not have time. Gender, age and nationality of 75 of the eligible but nonparticipating visitors were comparable to the recent visitors who were willing to participate. 9 out of 75 nonparticipants (12.0%) were HIV-infected as compared to 25 out of 197 (12.7%) participants included in the STI screening. Of interest, 65.3% of nonparticipants had not been sexually active in the past 6 months compared with 45.7% of the participants who consented to STI screening, *Χ* ^2^-test: p = 0.003.

Of the 197 participants who consented to STI screening, 141 (71.6%) were male and the majority were of Dutch nationality (84.2%). Their median age was 49.4 years (IQR 43.2–53.9). Characteristics of these participants at time of STI screening are presented in table 1. Of interest is the increase of reported methadone use, alcohol intake and the only use of cocaine as compared to all visitors at baseline. Blood was drawn from all participants and from 181 (91.9%) participants, urine or vaginal swabs were also tested. 190 (96.4%) participants were screened at the study site, and the other 7 (3.6%) followed the STI screening procedures at the STI clinic due to their high-risk STI profile.

Of 197 participants, 22 (11.2%) had evidence of previously treated syphilis. Of these 22 participants, 16 (72.7%) were female. None of the 197 participants was diagnosed with infectious syphilis. Of 181 samples screened at the study site for CT and NG, 3 participants (1.7%, all females and all HIV-negative) were diagnosed with CT. Two out of three participants were of non-Dutch ethnicity: Indonesian and Surinam. None of the 181 participants was diagnosed with NG.

### Determinants of Unprotected Sex Among Recent Visitors

Due to the low prevalence of STI among the 197 recent visitors, but a substantial prevalence of unprotected sex in longitudinal analyses, we decided to analyse determinants for unprotected sex among those who reported to be sexually active. 107 of the 197 (54.3%) recent visitors reported to have had vaginal or anal sex in the previous 6 months. In univariable analyses, we found that having a steady partner was significantly associated with unprotected sex compared to having a casual partner. HIV-positive status was negatively associated with unprotected sex. Multivariable analysis identified that having a steady partner (adjusted OR [aOR] 4.09, 95% CI 1.59–10.53) was positively associated with unprotected sex when compared to casual partnerships. HIV-infected participants were less likely to report unprotected sex (aOR 0.07, 95% CI 0.02–0.37) compared to HIV-uninfected participants (Table 2).

**Table 2:** Univariable and multivariable associations with unprotected sex among only sexually active recent visitors of the Amsterdam Cohort Studies (N = 107).

		Univariable		Multivariable	
	N (%)	OR (95% CI)	p-value	OR (95% CI)	p-value
Gender			0.10		
Male	71 (66.4)	1.00			
Female	36 (33.6)	2.03 (0.88–4.72)			
Age in years			0.33		
Per 10-year increase	107	1.31 (0.77–2.20)			
Ethnicity			0.22		
Dutch	87 (81.3)	1.00			
Non-Dutch	20 (18.7)	0.56 (0.22–1.43)			
Housing situation^a^			0.46		
Supervised	47 (43.9)	1.00			
Unsupervised	60 (56.1)	1.34 (0.62–2.92)			
Partner type^b^			0.003		0.001
Casual	42 (33.1)	1.00		1.00	
Steady	62 (48.8)	2.75 (1.17–6.47)		3.77 (1.46–9.75)	
Client	9 (7.1)	0.30 (0.06–1.45)		0.34 (0.07–1.63)	
CSW	14 (11.0)	0.41 (0.14–1.26)		0.32 (0.10–1.05)	
No. of sexual partners			0.75		
1	80 (74.8)	1.00			
>1	27 (25.2)	1.15 (0.50–2.66)			
HIV-status			0.012		
HIV-negative	98 (91.6)	1.00		1.00	0.002
HIV-positive	9 (8.4)	0.13 (0.03–0.64)		0.07 (0.02–0.37)	
Injection drug use			0.44		
No	98 (91.6)	1.00			
Yes	9 (8.4)	0.75 (0.18–3.06)			
Drug use^c^					
Only cocaine			0.60		
No	29 (27.1)	1.00			
Yes	78 (72.9)	0.80 (0.35–1.83)			
Only heroin			0.14		
No	52 (48.6)	1.00			
Yes	55 (51.4)	0.54 (0.24–1.21)			
Cocktail^d^			0.79		
No	103 (96.2)	1.00			
Yes	4 (3.7)	0.76 (0.10–5.91)			
Cannabis			0.77		
No	40 (37.4)	1.00			
Yes	67 (62.6)	1.12 (0.51–2.47)			
Other^e^			0.42		
No	58 (54.2)	1.00			
Yes	49 (45.8)	0.72 (0.33–1.57)			
Methadone on prescription			0.17		
No	52 (48.6)	1.00			
Yes	55 (51.4)	0.57 (0.25–1.26)			
Alcohol (any daily)			0.11		
No	39 (36.4)	1.00			
Yes	68 (63.6)	1.94 (0.86–4.36)			

CI, confidence interval; CSW, commercial sex worker; OR, odds ratio.

a Supervised: living in a hotel/pension, institutional care, lodging.

b Partner type: per individual more than 1 partner type possible.

c Includes injecting and noninjecting drug use; per individual, more than 1 type of drug use possible.

d Heroin and cocaine together.

e Includes benzodiazepines, amphetamines and barbiturates.

## Discussion

This study describes trends in HIV incidence, injecting drug use and sexual risk behaviour among DU of the ACS from 1986–2011. The major findings are declining trends in HIV incidence, injecting and sexual risk behaviour. In addition, STI screening performed among participants of the ACS with a study visit in 2010–2011 demonstrates a low STI prevalence. Although prevalence of unprotected sex is substantial, it is associated with having sex with a steady partner and, of interest, such prevalence is less in HIV-infected participants.

The decreasing trend in HIV incidence presented here is in line with other longitudinal studies and surveillance systems on drug using populations in high-income countries [2], [8], [9], [11], [23], [24]. However, many areas of the world report an increasing HIV-incidence rate among DU [6], [15], [25]–[27]. This epidemiologic discrepancy could be a result of inequalities in access to harm-reduction programmes and treatment services [15]. Coverage of HIV treatment and prevention services is highest in Western Europe, reaching 61% of the injecting DU [28]. As one of the first countries in Western Europe, the Netherlands initiated harm-reduction programmes in the 1980s [15]. The declining trend in the use of needle exchange, as observed in the ACS, was confirmed by a reduction in the absolute number of exchanged needles per calendar year in Amsterdam, which peaked in 1992 with 1,100,000 needles, whereas since 2007 about 150,000 needles per year were exchanged. A study to evaluate the effect of needle exchange programmes and opiate substitution therapy on HIV incidence among DU of the ACS found that the combination of these approaches was associated with a lower risk for acquiring HIV and hepatitis C infection [13]. Interestingly, phylogenetic analysis indicated that before 2002, 37 out of 47 cases who acquired HIV in the ACS were infected by subtype B virus strains specific for DUs, whereas after 2002 all four new HIV infections were unspecific for DUs. This might relate to the change in injecting risk behaviour [29].

In addition to the effect of harm-reduction programmes on reducing transmission through needles, injecting drug use seems to be out of fashion in the Netherlands [30]. According to data on young DU (aged 18–30) in Amsterdam, the proportion of individuals reporting a history of injection was 88% between 1985 and 1989 and declined to 31% between 2000 and 2004 [31]. On a broader level, new injecting DU constitute less than 10% of all injecting DU in 10 European countries [32]. The Netherlands appears to have the lowest rate of initiation of injecting among DU (2.1/100 PY) in Europe [33].

Another explanation for the declining trends in risk behaviour could be the aging population of the ACS. American studies support the finding that DU older than 50 years inject drugs [34] or share needles [35] less often than younger users. In addition, when comparing sexual risk behaviours among older and younger DU, older DU were less likely to have had sex in the past month [36]. Moreover, our analysis of the recent visitors revealed that a large number of participants reported zero sexual partners in the past 6 months. However, aging DU that do have sex still engage in high-risk sexual practices, such as inconsistent condom use [37], [38]. Selective loss to follow-up of high-risk participants could be another reason for the observed declines in risk behaviour over time. We demonstrated in a sensitivity analysis, however, that there were comparable declines among participants with a visit in 2010 as compared to the total DU population in the ACS. In addition, our findings are in line with national surveillance programmes showing that diagnoses of HIV, acute HBV and HCV infection are rarely reported in DU [39].

A previous study of the ACS in DU described that sources of HIV transmission changed from mainly related to injecting risk behaviour before 1996 to mainly related to unprotected sex after 1996 [7]. This change is of importance not only for DU populations but for others as well, since DU have the potential to serve as a bridge for sexual HIV transmission to the wider community [5], [27]. Of interest, in contrast to observations among men who have sex with men [40], no increase in sexual risk behaviour was found among HIV-infected DU of the ACS who initiated cART [41].

In a previous study among DU of the ACS between 1985 and 2005, we found a decline in HIV incidence and injecting, but not in sexual risk behaviour [7]. However, our data suggest a gradually decreasing proportion of any unprotected sex since 2004, accompanied by a low STI prevalence. Still, the prevalence of unprotected sex is substantial, but our results among the recent visitors demonstrate that unprotected sex is mainly done with a steady partner and is less common in HIV-infected participants. Furthermore, the recent visitors of the ACS show a low STI prevalence (2.5%), all diagnosed with CT. A CT screening in 2008 among young people (aged 15–29 years old) living in Amsterdam found a CT prevalence of 3.6% [42]. Data from drug treatment centres and other cohort studies from the United Kingdom and the United States all showed higher prevalences of CT and NG [43], [44]. This comparison of prevalences suggests that there is a low transmission rate of STI among DU in Amsterdam. These findings may indicate that there is no major risk for sexual HIV transmission among DU.

Due to the extension of the European Union, sex trafficking has become easier. A recent study among CSW who had migrated from eastern Europe to London found higher prevalences (although not significant) of HIV, CT, NG and syphilis in CSW from eastern Europe as compared to CSW from the United Kingdom [45]. In contrast, these migrants less commonly reported a history of drug use.

The current study has several limitations. First, our results can not be generalised to younger DU and those followed in regions with no or limited access to comprehensive harm-reduction programmes. Second, data on drugs and sexual risk behaviour were self-reported. Consequently, data could be influenced by socially desirable answers and therefore may underestimate true risk behaviour. However, STI screening and self-reported STI showed comparable prevalences, which suggests accuracy in reporting STI history, which has also been described before [46]. Third, to increase uptake for the STI screening among recent visitors we chose to use self-swabs. Unfortunately these self-swabs could not be analysed for TV. Other studies reported high prevalence of trichomoniasis among female DU, varying from 8.6% to 43% [43], [44], [46], [47]. We were only able to test for TV in the 7 high-risk participants. Furthermore, participants at high risk for STI and those who reported clinical symptoms were referred to the outpatient STI clinic of the Public Health Service of Amsterdam where more extensive testing occurred (including for TV).

Fourth, to confirm our findings regarding the low prevalence of STIs, the STI screening should be repeated and more data on STI prevalence among DU from outside our cohort is needed.

To conclude, we documented a continuing very low HIV-incidence rate accompanied by a low injecting risk behaviour among DU of the ACS. Prevalence of unprotected sex was substantial, but was mainly associated with having a steady partner and was less common in HIV-infected participants. Taken together with a low STI prevalence among the recent visitors, our findings indicate a low transmission risk of HIV and STI. These results suggest that DU no longer play a significant role in the spread of HIV in Amsterdam.
